# Promoter methylation might shift the balance of Galectin-3 & 12 expression in *de novo* adult acute myeloid leukemia patients

**DOI:** 10.3389/fgene.2023.1122864

**Published:** 2023-02-13

**Authors:** Magda Assem, Rady E. El-Araby, Ahmed A. Al-Karmalawy, Reem Nabil, Mohamed A. M. Kamal, Amany Belal, Heba I. Ghamry, Mohammed A. S. Abourehab, Mohammed M. Ghoneim, Mohammad Y. Alshahrani, Asmaa A. El Leithy

**Affiliations:** ^1^ Clinical Pathology Department, National Cancer Institute, Cairo University, Cairo, Egypt; ^2^ Division of Oral Biology, Department of Periodontology, Tufts University School of Medicine, Boston, MA, United States; ^3^ Central Lab, Theodor Bilharz Research Institute (TBRI), Ministry of Scientific Research, Cairo, Egypt; ^4^ Pharmaceutical Chemistry Department, Faculty of Pharmacy, Ahram Canadian University, Giza, Egypt; ^5^ Clinical Pathology Department, El-Hussein University Hospital, Al-Azhar University, Cairo, Egypt; ^6^ Medicinal Chemistry Department, Faculty of Pharmacy, Beni-Suef University, Beni-Suef, Egypt; ^7^ Department of Pharmaceutical Chemistry, College of Pharmacy, Taif University, Taif, Saudi Arabia; ^8^ Department of Home Economics, College of Home Economics, King Khalid University, Abha, Saudi Arabia; ^9^ Department of Pharmaceutics and Industrial Pharmacy, College of Pharmacy, Minia University, Minia, Egypt; ^10^ Department of Pharmaceutics, Faculty of Pharmacy, Umm Al-Qura University, Makkah, Saudi Arabia; ^11^ Department of Pharmacy Practice, College of Pharmacy, AlMaarefa University, Ad Diriyah, Saudi Arabia; ^12^ Research Center for Advanced Materials Science (RCAMS), King Khalid University, Abha, Saudi Arabia; ^13^ Department of Clinical Laboratory Sciences, College of Applied Medical Sciences, King Khalid University, Abha, Saudi Arabia; ^14^ College of Biotechnology, Misr University for Science and Technology (MUST), Giza, Egypt

**Keywords:** acute myeloid leukemia, promoter methylation, galectin-12, galectin-3, PCR

## Abstract

Acute myeloid leukemia (AML) was reported as the most common type of leukemia among adults. Galectins constitute a family of galactose-binding proteins reported to play a critical role in many malignancies including AML. Galectin-3 and -12 are members of the mammalian galectin family. To understand the contribution of galectin-3 and -12 promoter methylation to their expression, we performed bisulfite methylation-specific (MSP)-PCR and bisulfite genomic sequencing (BGS) of primary leukemic cells in patients with *de novo* AML before receiving any therapy. Here, we show a significant loss of *LGALS12* gene expression in association with promoter methylation. The lowest degree of expression was found in the methylated (M) group while the highest degree was in the unmethylated (U) group and the partially methylated (P) group expression lies in between. This was not the case with galectin-3 in our cohort unless the CpG sites analyzed were outside the frame of the studied fragment. We were also able to identify four CpG sites (CpG number 1, 5, 7& 8) in the promoter region of galectin-12; these sites must be unmethylated so that expression can be induced. As far as the authors know, these findings were not previously concluded in earlier studies.

## Introduction

Galectins an evolutionary conserved family are classified into three structural groups. Galectins are characterized by their ability to bind specific carbohydrates involved in a variety of cellular functions including cancer (Verkerke et al., 2022). This family of proteins is encoded by the *LGALS* genes family in humans and acts as an important recognizing factor towards cancer-associated glycoproteins (Yang et al., 2008). Their expression is very firmly controlled. Altered galectin expression is a hallmark of many cancer cells (Katzenmaier et al., 2017). Galectins can either be tumor promoters or suppressors based on their target cells (Kopitz et al., 2001). Furthermore, these family members had a prognostic influence on various types of malignancies including leukemias (Pena et al., 2014; Chetry et al., 2022). Galectin-12 structurally belongs to the galectins group which contains two homologous carbohydrate recognition domains (CRDs). Galectin-12 is a galectin family member encoded by the *LGALS12* gene with a partial expression in leukocytes and adipocytes (Xue et al., 2016). While, galectin-3 belongs to another galectin structural group (chimera group with only one CRD) was reported to play an important role in cancerous'-microenvironments, especially in acute myeloid leukemia (AML) (Burger, 2011; Icard et al., 2014; Evans and Calvi, 2015; Han et al., 2015; Pereira et al., 2015).

AML was reported as a disease with high heterogeneity, is also considered the highest hematologic malignancy in its fatality rate (Chen et al., 2013; Bray et al., 2018; Siegel et al., 2020). Despite the existence of various medications which were approved for patients with AML in the last few years, AML remains a condition with unmet medical needs (Bewersdorf and Abdel-Wahab, 2022).

It was reported that epigenetic dysregulation might contribute to the development of hematological malignancies, such as hypomethylation or increased methylation of the CpG islands in the promoter region of key genes (Gutierrez and Romero-Oliva, 2013). Epigenetic regulation of galectin-1,-3 &-7 was suggested to play a critical role in cancer progression (Chiariotti et al., 1994; Poirier et al., 2001; Margadant et al., 2012; Kim et al., 2013). Galectin-12 was shown to be silenced by DNA methylation in colorectal cancer (CRC) cell lines and primary samples (Xue et al., 2016; Katzenmaier et al., 2017). Moreover, previous studies showed that *LGALS3* promoter CpG islands were heavily methylated in the early stages of prostate adenocarcinoma (Ahmed and Bandyopadhyaya, 2015). Interestingly, another study reported that the average methylation degree of five CpG sites in the *LGALS3* gene regulatory region was significantly decreased in thyroid cancer tissues (Keller et al., 2013). Subsequently, the present study was conducted to address the analysis of methylation patterns in galectin-3 and -12 promoter regions in patients with *de novo* AML.

### Aim of the study

The present study aims to investigate to what extent the methylation patterns of *LGALS 3 & 12* promoter region are correlated with *LGALS 3 & 12* gene expression in adult AML patients.

### The research basis

This study was based on two previous findings:

First, the expression profiling of eight galectins was previously performed in adult AML. Interestingly, galectin-12 was the only galectin that showed a survival advantage in AML patients when overexpressed in peripheral blood (PB) (El Leithy et al., 2015). In addition, galectin-3 was almost exclusively downregulated in both PB& bone marrow (BM) (El Leithy et al., 2015; Abdelfattah et al., 2021).

Second, alteration of DNA methylation was frequently encountered in AML (Kim et al., 2013). Aberrant methylation of cytosine-5 at CpG sites were clustered in the gene promoter regions. Furthermore, DNA methylation was found to correlate with prognosis in AML (Toyota et al., 2001; Deneberg et al., 2010; Bacigalupo et al., 2013; Chattopadhyaya and Ghosal, 2022).

We conduct this study to find out whether reduced expression of galectins-3 and -12 are associated with methylation of CpG islands in their promoter region.

## Materials and methods

### Patient cohort and collection of samples

This cohort study included 171 samples; 73 BM and 98 PB samples from AML patients, 97 males (56.7%) and 74 females (43.3%) with a mean age of 38.5 years (SD 12.5) ranging from 18 to 63 years. All of these patients were presented to the inpatient clinic at the National Cancer Institute (NCI), Cairo University (CU), diagnosed between July 2012 to December 2017. Patients underwent routine laboratory investigations and imaging diagnoses and were classified according to the standard morphological and immunophenotyping (IPT) criteria. All samples were collected before treatment; patients were treated intensively with the standard protocol. Patients with acute promyelocytic leukemia (APL) were given All-trans retinoic acid (ATRA). Other FAB subtypes were given the 3 + 7 treatment protocol. Response to induction therapy was assessed between days 14 and 28 after induction therapy and none of them received hypomethylating therapy. Written informed consents were obtained from the patients or their legal guardians, and this study was approved by the ethical committee of NCI, CU, Egypt, and was in accordance with the 2011 Declaration of Helsinki (IRP Approval No. 201902012.4). The age and sex-matched group consists of 15 PB samples from healthy donors from the same hospital, and eight BM samples from volunteers for BM transplantation were selected as a control group.

### Methods

The present study sample size was estimated according to sample size estimation using the G*Power program (University of Düsseldorf, Düsseldorf, Germany) which is related to our previous work (El Leithy et al., 2015; Abdelfattah et al., 2021). The molecular assays were done on BM and PB whole white blood cell pellets; the initial blast cells median was 54% (ranging from 30% to 98%). It should be mentioned that the patient number is not the same in each technique depending on the availability of the samples in the lab. However, all samples were selected out of consecutive cohorts which meet the study eligibility criteria. That’s to say methylation analysis and gene expression were done on the same patients, after normalization of the genes expression to healthy donors, while the methylation analysis was assessed only in the patient cohort. This was done to evaluate the effect of the genes promotor methylation on the corresponding gene expression in the same samples.

### RNA extraction and quantitative reverse transcriptase-polymerase chain reaction (qRT-PCR)

The total cellular RNA from the number of BM and PB blood samples was purified to profile and associate the expression of *LGALS 3 & 12*. This was done using Invitrogen™ TRIzol™ Reagent (Invitrogen™, Thermo Fisher Scientific). Afterwards it was reversely transcribed to cDNA using Applied Biosystems™ (Thermo Fisher Scientific). The qRT-PCR was performed using Applied Biosystems PowerUp™ SYBR™ Green Master Mix (Thermo Fisher Scientific) according to the manufacturer’s instruction on Applied Biosystems Step One™ Real-Time PCR System (Thermo Fisher Scientific). The sequences of the forward and reverse primers related to *LGALS3*, *LGALS12,* and *GAPDH* are shown in Table 1. The primers were designed using the Primer-BLAST tool available on the NCBI website (https://www.ncbi.nlm.nih.gov/tools/primer-blast/).

**TABLE 1:** The sequences of the forward and reverse primers for LGALS 3, 12, & GAPDH.

Gene	Primer sequence
*LGALS3*	
Forward	ATG​GCA​GAC​AAT​TTT​TCG​CTC​C
Reverse	GCC​TGT​CCA​GGA​TAA​GCC​C
*LGALS12*	
Forward	GCC​TGG​GCA​GGT​CAT​CAT​AG
Reverse	GAG​TTC​TGT​CTG​CGA​AGG​AGG
*GAPDH*	
Forward	CTG​GGC​TAC​ACT​GAG​CAC​C
Reverse	AAG​TGG​TCG​TTG​AGG​GCA​ATG

### Differential DNA methylation analysis

Genomic DNA extraction and bisulfite conversion: Genomic DNA was isolated from 52 BM samples using G-spin™ Total DNA Extraction Mini Kit (LiliF Diagnostic Products). Then genomic DNA was bisulfite-modified using Thermo Scientific™ EpiJET™ Bisulfite Conversion Kit (Thermo Fisher Scientific) according to the manufacturer’s instruction. Subsequently, to investigate the effect of the DNA methylation on galectin-3&12 expressions in AML patients, methylation was analyzed by two methods on two separate cohorts as follows: 1- methylation-specific (MSP)-PCR for *LGALS3 & 12* in 24 cases and 2- bisulfite genomic sequencing (BGS) for *LGALS12* in 28 cases.

### Methylation-specific (MSP)-PCR for *LGALS3 & 12*

**FIGURE 1:**
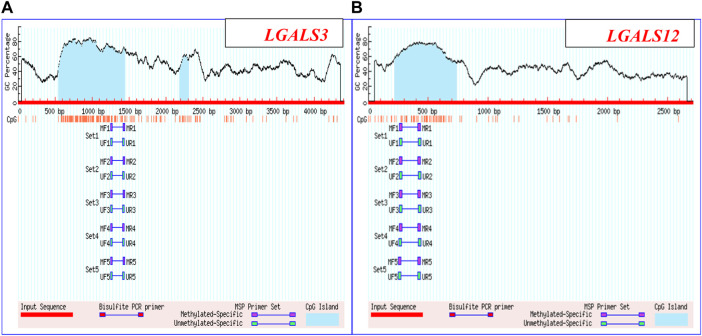
*LGALS3 & 12* promoter regions: **(A)** Promoter region of *LGALS3*: Chromosome 14: 55128400-55132801 & **(B)** Promoter region of *LGALS12*: Chromosome 11: 63490635-63492346.

The methylation pattern of *LGALS3 & 12* genes promoter region was carried out on 24 subjects. For performing MSP, four pairs of primers were applied and specified for the methylated and un-methylated targeted sequences as shown in Figures 1A, B. Primer sequences are shown in Table 2. All of the MSP primers were designed using the MethPrimer design tool (http://www.urogene.org/methprimer/). Then the specificity of the primers was applied using the “by search” tool.

**TABLE 2:** Profile of primers used in MSP.

Gene		Primer	Sequence	Product size (bp)	Tm (^⸰^C)
***LGALS3***	**Methylated**	Forward	AGT​AAG​TTT​TAT​TCG​GTG​ACG​AGT​C	192	57
		Reverse	TAT​ACA​ATC​CTA​AAA​AAT​CCC​TTC​G		
	**Unmethylated**	Forward	AAG​TTT​TAT​TTG​GTG​ATG​AGT​TGT	187	58
		Reverse	TAC​AAT​CCT​AAA​AAA​TCC​CTT​CAC​T		
***LGALS12***	**Methylated**	Forward	GGT​ATA​GTT​GAA​CGT​TTG​AGC​GT	177	59
		Reverse	TAC​AAA​ACC​TAA​AAA​CCG​ACG​AA		
	**Unmethylated**	Forward	GGG​GTA​TAG​TTG​AAT​GTT​TGA​GTG​T	181	60
		Reverse	CCT​ACA​AAA​CCT​AAA​AAC​CAA​CAA​A		

Amplification was carried out in a thermocycler. For controlling and optimizing the MSP reactions, the EpiTect® PCR Control DNA Kit (Qiagen, Hilden, Germany) was used according to the manufacturer’s instructions. Methylation in this case is considered based on the amplification shown by samples with M primer as well as with both M and U primers. Samples showing amplification with only U were considered as unmethylated. Bands corresponding to methylated partners determined complete methylation. Partial methylation is determined by bands corresponding with both M and U primers.

### Genomic DNA methylation sequencing of galectin-12 as a validation method for its methylation pattern

The second cohort included twenty-eight BM samples at diagnosis; they were enrolled for targeted bisulfite sequencing. MethPrimer software, (http://www.urogene.org/cgi-bin/methprimer/methprimer.cgi), was used for designing a specific set of primer pairs (M13-tailed PCR and *LGALS12* primers) shown in Table 3 that binds only to bisulfite-modified DNA. This analysis was focused on a genomic region rich in CpG islands (by using DBCAT software, http://dbcat.cgm.ntu.edu.tw), containing 11 CpGs at nt −445 to nt −213 upstream of exon 1 in the predicted promoter region of *LGALS12*. This region also includes binding site for the transcription factor well known as SP1 that binds to GC-rich motives of many promoters (Katzenmaier et al., 2017).

**TABLE 3:** Profile of primer used for CpG methylation analysis (used in BGS).

	Primer used for CpG methylation analysis
**M13 tailed**	
Forward	GTAAAACGACGGCCAG
Reverse	CAGGAAACAGCTATGAC
***LGALS12* gene**	
Forward	GAG​TTT​TAG​GGG​GTT​GTA​AAA​TTT
Reverse	AAT​CTT​ACT​CTC​TTA​CCA​AAC​TAC​A

PCR reaction was carried out on Applied Biosystems™ Veriti™ 96-Well Fast Thermal Cycler (Thermo Fisher Scientific). Sequencing was carried out using BigDye™ Terminator v3.1 Cycle Sequencing Kit (Thermo Fisher Scientific) according to the manufacturer’s instructions after the cycle sequencing. Products were purified using The BigDye® XTerminator™ Purification Kit (Thermo Fisher Scientific). Applied Biosystems™ 3500 XL Genetic Analyzer (Thermo Fisher Scientific) carried out targeted automatic bisulfite sequencing reaction and sequencing data analysis.

### Statistical analysis

The data were analyzed using a statistical package for social science ‘IBM SPSS Statistics for Windows, version 26 (IBM Corp., Armonk, N.Y., United States). Continuous normally distributed variables were represented as mean ± SD with a 95% confidence interval, while non-normal variables were summarized as median with 25 and 75 percentiles, and using the frequencies and percentage for categorical variables; a *p-value* < 0.05 was considered statistically significant. To compare the means of normally distributed variables between groups, the student’s t-test was performed, while the Mann-Whitney U test was used in non-normal variables. χ^2^ test was used to determine the distribution of categorical variables between groups. Pearson correlation was done to measure if there was any linear association between *LGALS3 & 12* gene expression. For MSP-PCR results, univariate analysis was conducted to determine the prognostic performance of each studied biomarker. In addition, Survival analysis was performed using a Kaplan-Meier test.

## Results

### *LGALS3 & 12* expressions in AML patients

**FIGURE 2:**
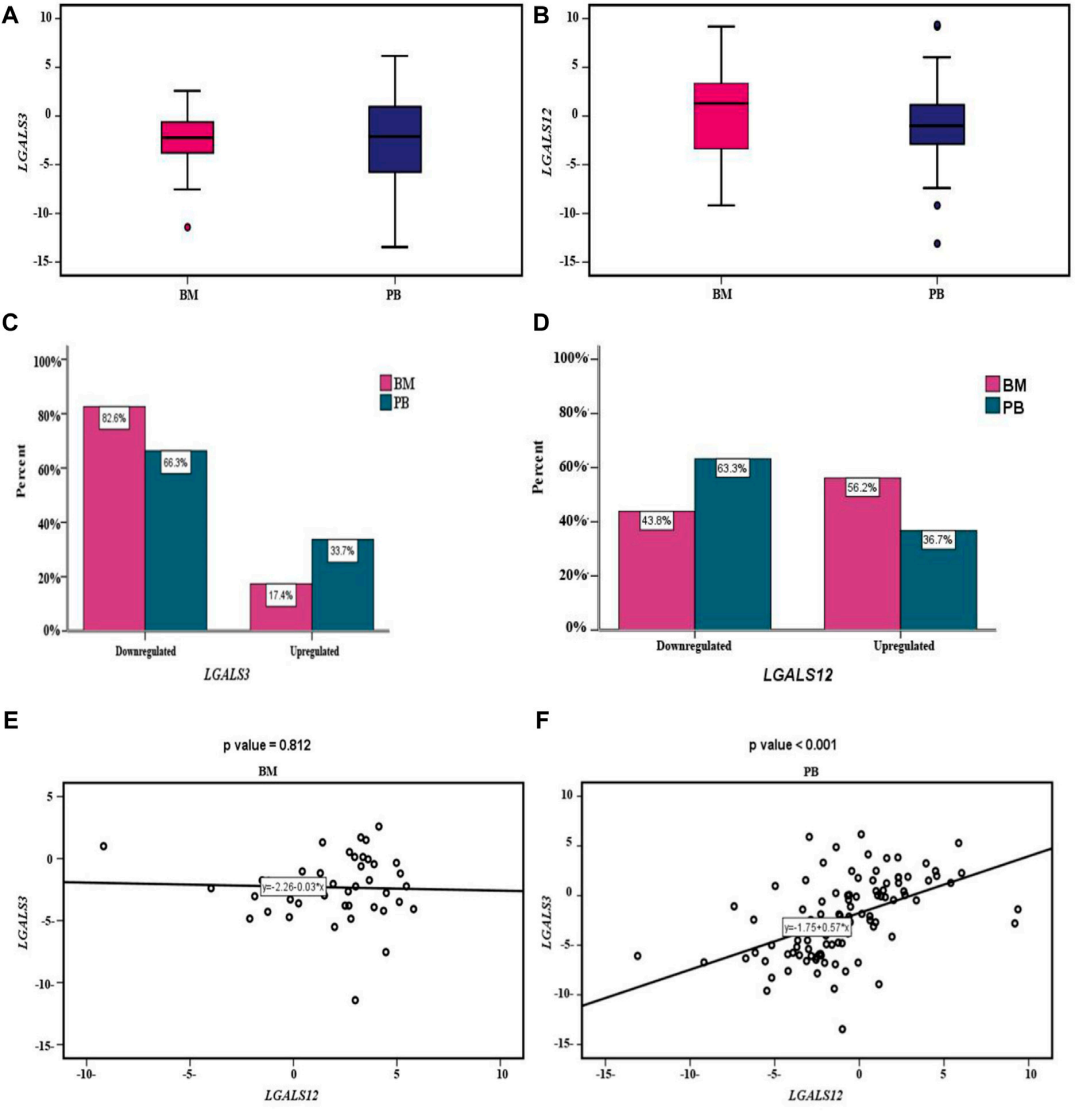
*LGALS3 & 12* expressions in AML patients: **(A)** Box plot showing differential expression of the *LGALS3* gene in PB & BM; the X-axis represents the *LGALS3* gene and the Y-axis shows BM and PB cohorts. **(B)** Box plot showing differential expression of the *LGALS12* gene in PB & BM; the X-axis represents the *LGALS12* gene and the Y-axis shows BM and PB cohorts. **(C)** Cluster bar showing association between the *LGALS3* gene expression in PB & BM; the X-axis represents the percent and the Y-axis shows the *LGALS3* gene expression in BM and PB cohorts. **(D)** Cluster bar showing association between *LGALS12* gene expression in PB & BM; the X-axis represents the percent and the Y-axis shows the *LGALS12* gene expression in BM and PB cohorts. **(E)** Scatter plot showing the correlation between *LGALS3*&*12* genes expression within BM. The X-axis represents the *LGALS3* gene expression and the Y-axis shows the *LGALS12* gene expression. **(F)** Scatter plot showing the correlation between *LGALS3*&*12* genes expression within PB; the X-axis represents the *LGALS3* gene expression and the Y-axis shows the *LGALS12* gene expression. Abbreviations: BM (Bone marrow); PB (Peripheral blood).

The profiling of *LGALS3 & 12* in both PB and BM of the AML patients showed differential expression as shown in (Figures 2A,B). The *LGALS3* gene expression in PB and BM showed an association in the gene down-regulation in BM more than in PB (82.6% vs*.* 66.3% *p-value* = 0.044). Where, *LGALS12* gene expression exhibited more downregulation in PB than in BM (63.3% vs*.* 43.8% *p-value* = 0.012) (Table 4; Figures 2C,D).

**TABLE 4:** Expression profile of *LGALS3 & 12* in both PB and BM of the AML patients.

***LGALS3***	**BM (n = 46)**	**PB (n = 98)**	***p-value***
Upregulated	8 (17.4%)	33 (33.7%)	0.044
Downregulated	38 (82.6%)	65 (66.3%)	
***LGALS12***	**BM** (**n= 73)**	**PB** (**n= 98)**	
Upregulated	41 (56.2%)	36 (36.7%)	0.012
Downregulated	32 (43.8%)	62 (63.3%)	

*p-value* was performed using the χ2 test.

By studying the correlation between *LGALS3 & 12* genes expression in PB and BM we found that; no statistically significant correlation was found between both genes’ expression in BM (r = −0.036 and *p-value* = 0.812). Conversely in the PB, a statistically significant moderate positive correlation was found (r = 0.5 and *p-value* < 0.001), Figures 2E,F.

### The methylation pattern of *LGALS3 & 12* gene promoter region

(MSP)-PCR for *LGALS3* exhibited that one (4%) out of 24 examined subjects were completely methylated (M), 5 (21%) were partially methylated (P) and 18 (75%) were unmethylated (U). While *LGALS12* promoter region showed that 5 (21%) were completely M out of 24 examined subjects, 12 (50%) were P and 7 (29%) were U.

Because most of the studied group by MSP-PCR exhibited an unmethylated pattern (75%) for the *LGALS3* promoter region, BGS as a validation step was carried out only for *LGALS12*.

### Relation of MSP of *LGALS 12* with clinicolaboratory data

In this study, we compared all the clinical characteristics of patients with *LGALS12* methylation patterns (Table 5). The results showed that there was a positive association with the adverse genetic risk in P and M cases when compared with the U group (*p-value* = 0.05 and 0.001, respectively). Furthermore, there was a positive risk for M with Odd Ratio (OR) = 4 (*p-value* = 0.01). Likewise, there was a positive association with the adverse genetic risk in M cases when compared with the P group (*p-value* < 0.001). In addition, there was a positive risk for the M group with OR = 2.00 (*p-value* = 0.05).

**TABLE 5:** Relation of the methylation pattern of galectin 12 and clinicolaboratory data of the studied group.

		Galectin 12 methylation pattern						Risk assessment					
		Un-methylated n = 7	Partially methylated n = 12	Completely methylated n = 5	*p-value*			P & U		M & U		M & P	
					P & U	M & U	M & P	OR (95%C.I)	*p-value*	OR (95%C.I)	*p-value*	OR (95%C.I)	*p-value*
**Age**		40.4 ± 12.9	39.0 ± 14.4	37.2 ± 6.7	0.8	0.6	0.3	0.99 (0.92–1.07)	0.8	0.97 (0.86–1.09)	0.6	0.99 (0.96–1.02)	0.4
**Sex**	**Female**	4 (57.1%)	5 (41.7%)	3 (60.0%)	0.08	0.6	0.07	1.87 (0.28–12.31)	0.5	0.89 (0.09–9.16)	0.9	0.48 (0.22–1.01)	0.08
	**Male**	3 (42.9%)	7 (58.3%)	2 (40.0%)									
**HB**		6.8 ± 3.3	8.4 ± 2.1	6.1 ± 2.7	0.4	0.8	0.2	1.34 (0.74–2.45)	0.3	0.91 (0.53–1.55)	0.7	0.57 (0.41–0.79)	0.3
**TLC**		14.9 (6.7–140.0)	170.5 (94.1–440.0)	80.8 (3.6–158.8)	0.06	0.8	0.2	1.02 (0.99–1.04)	0.2	1.00 (0.99–1.02)	0.6	0.99 (0.98–0.99)	0.3
**PLT**		19.0 (15.5–104.3)	29.0 (28.3–35.5)	17.0 (9.3–36.0)	0.2	0.5	0.2	0.99 (0.94–1.03)	0.5	0.98 (0.92–1.04)	0.4	0.88 (0.83–0.94)	0.3
**PB blast %**		56.3 ± 32.7	71.7 ± 22.3	70.5 ± 34.1	0.4	0.7	0.9	1.03 (0.97–1.08)	0.3	1.02 (0.97–1.07)	0.5	1.00 (0.98–1.02)	0.8
**BM blast %**		77.8 ± 14.5	70.2 ± 11.6	61.0 ± 28.1	0.4	0.3	0.6	0.94 (0.83–1.07)	0.4	0.96 (0.89–1.04)	0.3	0.97 (0.95–1.00)	0.06
***FLT3-ITD***	**wild**	6 (85.7%)	9 (75.0%)	4 (80.0%)	0.07	0.2	0.2	2.00 (0.17–24.07)	0.6	1.50 (0.07–31.57)	0.8	0.75 (0.30–1.85)	0.5
	**Mutant**	1 (14.3%)	3 (25.0%)	1 (20.0%)									
**Genetic risk**	**Normal**	4 (57.1%)	7 (58.3%)	2 (40.0%)	0.8	0.06	0.01*	1.75 (0.51–5.98)	0.4	0.57 (0.31–1.06)	0.07	0.29 (0.16–0.50)	<0.001**
	**Intermediate**	3 (42.9%)	4 (33.3%)	1 (20.0%)	0.08	0.01*	0.05*	1.33 (0.30–5.96)	0.7	0.33 (0.15–0.74)	0.01*	0.25 (0.12–0.54)	<0.001**
	**Adverse**	0 (0.0%)	1 (8.3%)	2 (40.0%)	0.05*	<0.001**	<0.001**	‒	‒	4.00 (1.34–11.96)	0.01*	2.00 (0.86–4.67)	0.05*
**Hepatomegaly**	**No**	6 (85.7%)	12 (100.0%)	3 (60.0%)	0.07	0.01*	0.01*	‒	‒	4.00 (1.50–10.66)	0.01*	‒	‒
	**Yes**	1 (14.3%)	0 (0.0%)	2 (40.0%)									
**Splenomegaly**	**No**	6 (85.7%)	12 (100.0%)	3 (60.0%)	0.07	0.01*	0.01*	‒	‒	4.00 (1.50–10.66)	0.01*	‒	‒
	**Yes**	1 (14.3%)	0 (0.0%)	2 (40.0%)									
**LNs**	**No**	7 (100.0%)	10 (83.3%)	2 (40.0%)	<0.001**	<0.001**	<0.001**	‒	‒	‒	‒	7.50 (3.27–17.19)	<0.001**
	**Yes**	0 (0.0%)	2 (16.7%)	3 (60.0%)									
**IPT**	**Mono**	4 (57.1%)	7 (58.3%)	2 (40.0%)	0.8	0.01*	0.01*	1.75 (0.51–5.98)	0.4	0.50 (0.27–0.91)	0.02*	0.29 (0.16–0.50)	<0.001**
	**Myelo**	2 (28.6%)	4 (33.3%)	2 (40.0%)	0.3	0.2	0.06	2.00 (0.37–10.92)	0.4	1.00 (0.50–2.00)	0.9	0.50 (0.27–0.91)	0.07
	**Myelomono**	1 (14.3%)	1 (8.3%)	1 (20.0%)	0.1	0.2	0.05*	1.00 (0.06–15.99)	0.9	1.00 (0.38–2.66)	0.9	1.00 (0.38–2.66)	0.9
**CR**	**No**	5 (71.4%)	8 (66.7%)	4 (80.0%)	0.3	0.1	0.08	1.25 (0.16–9.54)	0.8	0.63 (0.24–1.64	0.3	0.50 (0.21–1.21)	0.1
	**Yes**	2 (28.6%)	4 (33.3%)	1 (20.0%)									
**Death**	**No**	3 (42.9%)	6 (50.0%)	2 (40.0%)	0.2	0.6	0.04*	0.75 (0.11–4.90)	0.8	1.12 (0.49–2.57)	0.8	1.50 (0.71–3.17)	0.3
	**Yes**	4 (57.1%)	6 (50.0%)	3 (60.0%)									

*p-value* was performed using χ2 test, (*) *p-value* < 0.05 is significant, (**) *p-value* < 0.001 is highly significant. Abbreviations: M (methylated); P (partially methylated), U (unmethylated), HB (hemoglobin); TLC (Total leukocyte count); PLT (platelets); BM (Bone marrow); PB (Peripheral blood); LNs (lymph nodes), IPT (immunophenotyping); CR (complete remission).

On the other hand, there was a negative association with intermediate genetic risk in M cases when compared with the U group (*p-value* = 0.01) with protection from the M with OR = 0.33 (*p-value* = 0.01). Similar, results were given when the M group was compared with the P group (*p-value* = 0.05), OR = 0.25 (*p-value* < 0.001).

Regarding the hepatomegaly and splenomegaly associations, the results showed that there was a positive association with M in the cases that had organomegaly of the liver and spleen when compared with the U and/or P groups (*p-value* = 0.01 and 0.01, respectively).

In addition, the results showed that there was a positive association with lymph nodes (LNs) involvement in P cases and M cases when compared with the U group (*p-value* = 0.001 and 0.001, respectively). Likewise, there was a positive association with lymph node-positive in M cases when compared with the P group (*p-value* ***<*** 0.001). Moreover, there was a positive risk for M with OR = 7.50 (*p-value* = 0.01).

Regarding the immunophenotyping (IPT), the results showed that there was a positive association with the myelomono IPT in M cases when compared with the P group (*p-value =* 0.05). We found that there was a negative association with mono IPT in M cases when compared with the U group (*p-value* = 0.01) with protection from the M (OR = 0.50 (*p-value =* 0.02)). Similar results were given when the M group was compared with the P group, OR = 0.29 (*p-value* ***<*** 0.001).

Besides, the results showed a positive association with the mortality rate in M cases when compared with the P group (*p-value =* 0.04).

### Impact of methylation pattern of *LGALS12* on its gene expression and patients’ overall survival

**FIGURE 3:**
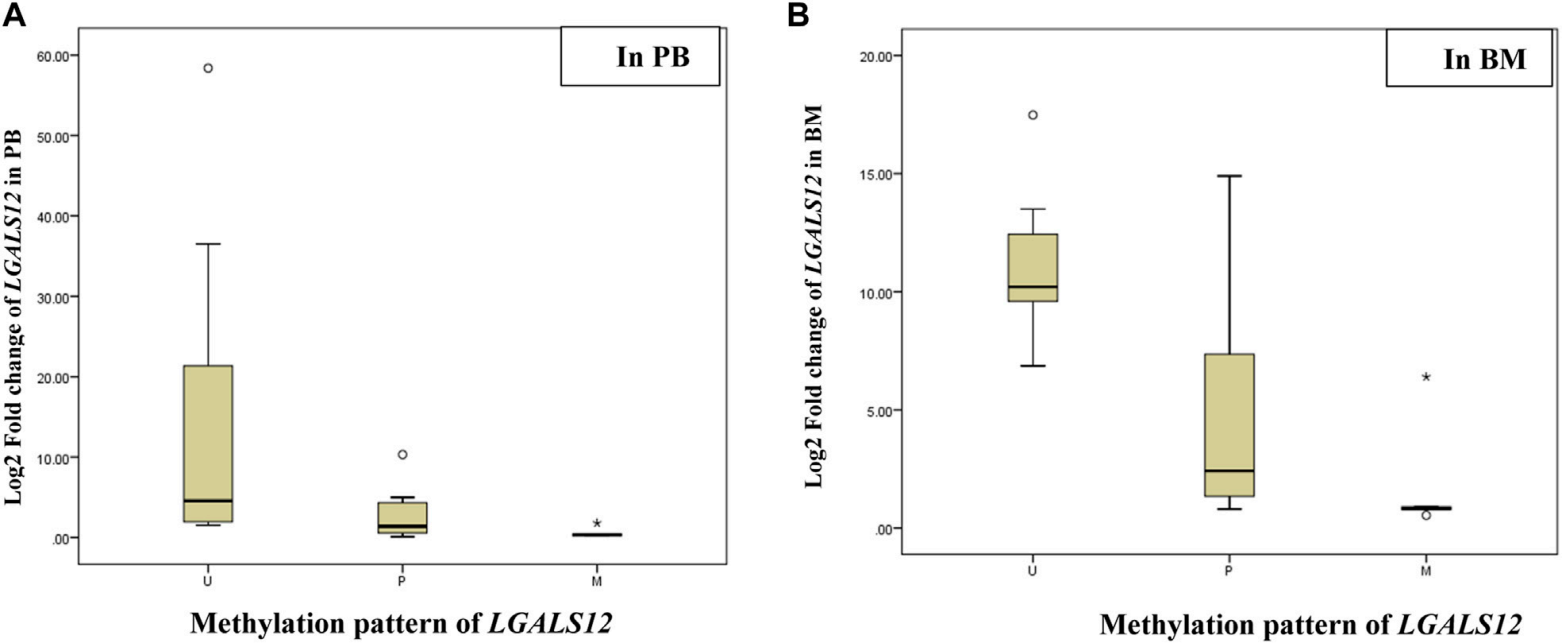
Log2 fold change of the *LGALS12* gene expression associated with the methylation pattern of its promoter region. Abbreviations: M (methylated); P (partially methylated), U (unmethylated), BM (Bone marrow); PB (Peripheral blood). **(A)** Box plot showing Log2 fold change of the *LGALS12* gene expression associated with the methylation pattern of its promoter region in PB; the X-axis represents the methylation pattern of *LGALS12* gene and the Y-axis shows Log2 fold change of the *LGALS12* gene expression in PB cohort. **(B)** Box plot showing Log2 fold change of the *LGALS12* gene expression associated with the methylation pattern of its promoter region in BM; the X-axis represents the methylation pattern of *LGALS12* gene and the Y-axis shows Log2 fold change of the *LGALS12* gene expression in BM cohort.

The association results showed a positive association between *LGALS-12* downregulation and an increasing incidence of methylation patterns. *LGALS-12* was downregulated in P or M cases when compared to U cases in PB samples (*p-value =* 0.03 and 0.01, respectively) and BM samples (*p-value =* 0.003 and 0.01, respectively). Moreover *LGALS-12* was downregulated in M cases when compared to P cases in PB samples (*p-value =* 0.01) and BM samples (*p-value =* 0.01), (Table 6; Figure 3).

**TABLE 6:** The association between the methylation pattern of *LGALS12* and its gene expression.

*LGALS12* expression	*LGALS12* methylation pattern						Risk assessment					
	U (n = 7)	P (n = 12)	M (n = 5)	*p- value*			P & U		M & U		M & P	
				P & U	M & U	M & P	OR (95%C.I)	*p- value*	OR (95%C.I)	*p- value*	OR (95%C.I)	*p- value*
**PB**	4.6 (1.8–36.5)	1.4 (0.5–4.5)	0.4 (0.2–1.1)	0.03*	0.01*	0.01*	0.88 (0.78–0.99)	0.04*	0.03 (0.001–0.19)	<0.001**	0.38 (0.23–0.65)	<0.001**
**BM**	10.2 (9.6–13.5)	2.4 (1.3–9.8)	0.8 (0.7–3.7)	0.03*	0.003**	0.01*	0.76 (0.68–0.86)	0.04*	0.78 (0.54–0.92)	0.01*	0.81 (0.69–0.94)	0.01*

*p-value* was performed using χ2 test, (*) *p-value* < 0.05 is significant, (**) *p-value* < 0.001 is highly significant. Abbreviations: M (methylated); P (partially methylated), U (unmethylated), BM (Bone marrow); PB (Peripheral blood); BM (Bone marrow); PB (Peripheral blood).

The linear regression analysis results confirmed the abovementioned association results. As the OR of the P or M cases was compared with U cases in PB samples = 0.88 (*p-value =* 0.04), 0.03 (*p-value <* 0.001) and OR of BM samples = 0.76 (*p-value =* 0.04), and 0.78 (*p-value =* 0.01), respectively. In addition, OR of M cases was compared with P cases in PB samples = 0.38 (*p-value <* 0.001) and BM samples = 0.81 (*p-value =* 0.01), Table 6.

**FIGURE 4:**
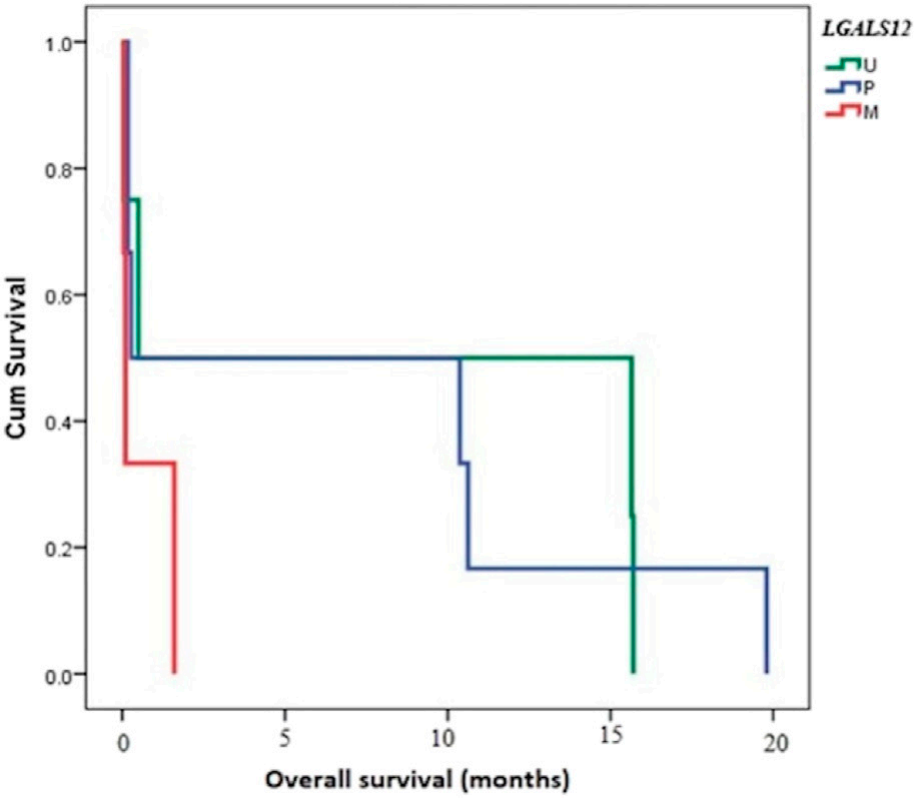
Overall survival (OS) and its relation to methylation pattern of *LGALS12*. Abbreviations: M (methylated); P (partially methylated), U (unmethylated), BM (Bone marrow); PB (Peripheral blood).

Follow-up of cases was done for 20 months. The median follow-up time was 3.75 months (ranging from 0.07 to 19.28 months). The OS of AML patients was measured from the date of diagnosis until the date of death or censoring for patients alive at the last follow-up. Studying the relation of OS and *LGALS-12* methylation pattern showed a very close time to death in M cases when compared with U cases (*p-value* < 0.001). The same results were given, in the case of M group compared with P (*p-value* < 0.001), Figure 4.

### Galectin-12 methylated CpG islands

**FIGURE 5:**
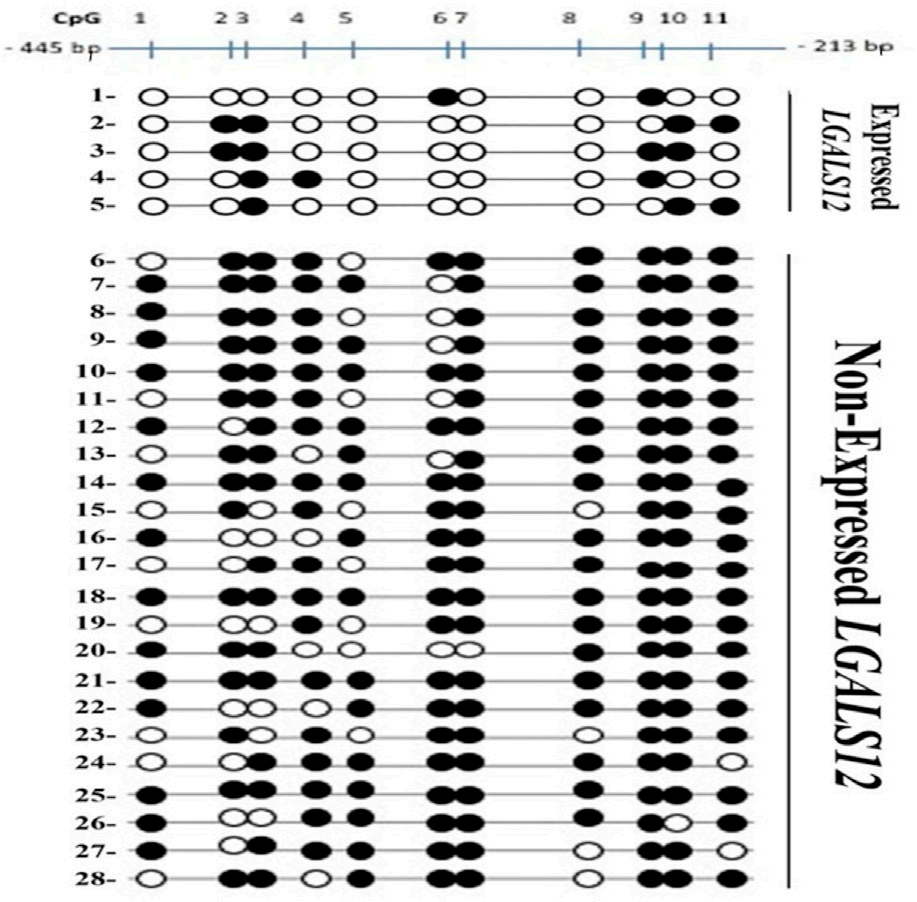
Bisulfite Genomic Sequencing of the promoter region of *LGALS12*. Methylation status of CpGs of Gelactin-12 promoter. CpGs are shown as either black (methylated) or white (unmethylated) circles and are numbered by Roman numerals.

The total number of CpG sites analyzed was 308 in 28 AML patients (Figure 5). Patients were divided based on their expression pattern into 2 groups. In the first non-expressed group (23 patients), the total number of CpG sites studied in the non-expressed group was 253 mostly methylated 203; the unmethylated sites were only 50/353 (19.8%). Second, expressed group (five patients), the total number in this expressed group was 55 mostly unmethylated 39/55 (70.1%), and 16 sites were methylated.

Analysis of methylation pattern per patient revealed that:

In the non-expressed group (23 patients) five patients had all eleven (11/11) CpG sites methylated. Three patients had 10/11 CpG sites methylated. Two patients had 9/11 sites methylated. Nine patients had 8/11 CpG sites methylated and four patients had 7/11 CpG sites methylated.

In the expressed group (five patients) one patient had 2/11 CpG sites methylated, two patients had 3/11 CpG sites methylated and two patients had 4/11 CpG sites methylated. None of the studied AML patients showed 5/11 or 6/11 methylated sites.

Analysis of methylation pattern per CpG locus revealed that:

In the unexpressed group; CpG number 1 9/23 were unmethylated, CpG number 2 &5 8/23 were unmethylated, CpG number 3 & 6 6/23 were unmethylated, CpG number. 4 5/23 were unmethylated, CpG number 7& 10 1/23 were unmethylated, CpG number 8 4/23 were unmethylated and CpG number nine all were methylated. CpG number 11 2/23 were unmethylated.

In the expressed group; CpG number 1, 5, 7 & 8 were all unmethylated, CpG number 4 & 6 were unmethylated in 4/5, CpG number 2 &11 were unmethylated in 3/5, CpG number 9&10 were unmethylated in 2/5 and CpG number 3 was unmethylated in 1/5.

## Discussion

The stimulator to leukemogenesis is a result of coordinated alterations in epigenetic regulation including methylation. Both gene-specific and global methylation patterns could predict outcomes in patients in AML (Deneberg et al., 2010). Previous studies showed that abnormal expression of some galectins correlates with tumor growth, cell migration, invasion, tumor aggressiveness, metastasis, recurrence, and poor prognosis in various cancers including leukemia (Ahmed et al., 2009; Bacigalupo et al., 2013; Ruvolo, 2019).

In the present study, despite the fact there was a statistically significant association between *LGALS3 &12* genes expression in both PB and BM, there was no significant correlation between the expression in BM & PB of these two galectins in our previous study (Abdelfattah et al., 2021). This novelty could be due to the relatively large sample size studied here. In the current study, *LGALS3 & 12* were both downregulated (66.3% & 63.3% respectively) in PB, and *LGALS3* was even more downregulated in the BM (82.6%) while *LGALS12* was less downregulated in the BM (43.8%). This could possibly be attributed to the fact that its expression in the BM is not only in leukocytes but also is in adipocytes (Xue et al., 2016). Galectin-12 was found to be preferentially expressed by human adipocytes and functions as an intrinsic negative regulator of lipolysis. In addition to its important function as an intracellular regulator of sebocyte proliferation (Tsao et al., 2022).

The study shows that, *LGALS3* gene expression in PB and BM was mostly downregulated but contrary to our findings, Cheng et al. (2013) demonstrated that a higher bone marrow *LGALS3* protein expression was an independent unfavorable prognostic factor for OS in patients with AML in Taiwan. This difference could be attributed to racial disparity and ethnic variation (Balan et al., 2008). Unlike our finding Ruebel et al. (2005) found that *LGALS3* gene expression is decreased upon methylation in its promoter region in some pituitary tumors, however, their study was only restricted to cell lines for different types of cancer.

In fact, galectin-3 functionality depends on its subcellular localization, whether nuclear, cytoplasmic, cell surface, or circulating. Whereby cytoplasmic and circulating galectin-3 provide the most cell growth promotion (Newlaczyl and Yu, 2011). Ruvolo (2019) elaborated on the survival advantage induced by galectin-3 in the leukemic niche. He & Farhad *et al.* (2018) showed the role of MSC-derived galectin 3 in the AML microenvironment (Farhad et al., 2018; Ruvolo, 2019).

Herein, this study examined the methylation pattern of galectin-3 using MSP-PCR which resulted in most of the cases being unmethylated (18/24), six cases were partially methylated and only one case was completely methylated, despite the predominant low expression pattern of *LGALS3*. The role of *LGALS3* expression pattern in carcinogenesis was extensively investigated in previous studies (Ebrahim et al., 2014). Silencing of galectin-3 expression by methylation of its promoter was associated with early stages of prostate cancer (Cummings et al., 2022). Our study outcome however did not give the same results in AML adult patients, unless, methylated CpG sites might be present outside the studied region.

Regarding *LGALS12*, we analyzed the methylation pattern of its promoter region by 2 methods. In the first cohort (MSP-PCR), most of our cases 12/24 (50%) were in the P category. This could be attributed to contamination by normal cells as reported previously (Quesnel et al., 1998), or by the fact that methylation in the CpG islands was not consistent in all AML samples (Galm et al., 2005). Our validation cohort supports the second notion since the percentage of methylation in the 11 CpG sites varied among patients ranging from 100% (11/11) to 7/11 but not less than seven sites methylation in patients who did not express galectin-12. At least seven CpG loci out of the eleven were methylated in the non-expressed group. In addition, we identified four CpG sites (1, 5, 7& 8) in the promoter region of galectin-12. All four must be unmethylated so that *LGALS12* expression can be induced. To the authors’ knowledge; it is the first time to report such novelty in AML patients, an assumption that needs to be proven.

Regarding the clinical data of the patients, in the same institution, a previous study showed a significant association between splenomegaly and a relatively higher *LGALS3* expression (Abdelfattah et al., 2021). Galectin-3 is known to be a powerful chemoattractant for monocytes, macrophages, and dendritic cells (Sano et al., 2000; Hsu et al., 2009). Thus we can postulate that its relatively higher expression in the bone marrow might attract the cells in numerous numbers which will be successfully drained into the spleen causing its expansion.

Here, our results showed a significant association between the promoter methylation status of galectin-12 & splenomegaly, hepatomegaly and lymphadenopathy. All cases with lymph nodes (LNs) enlarged were methylated 5/5 either partially or completely methylated (*p-value* < 0.001) for each. None of LNs enlargement was in the unmethylated group (*p-value* < 0.001). Higher expression of *LGALS12* was shown to cause cell cycle arrest and apoptosis; probably causing shrinkage of the spleen and lymph nodes (Yang et al., 2001). Interestingly all 3 cases in the adverse genetic risk group were either partially (one case) or completely methylated (2 cases) in galectin-12 (*p-value* < 0.001) while none were in the unmethylated group. Also, it is in accordance with Farzaneh *et al* (2022), they showed that methylation as a biological process influences gene expression by affecting the promoter activity in colorectal cancer (Ghadiri Moghaddam et al., 2022). We found *LGALS12* expression in the bone marrow only is border line significantly associated with AML-M4 compared to the other AML subtypes (*p-value* = 0.05) (data not shown), however, our methylation analysis showed a statistically significant association between complete methylation and unmethylation in the mono subtype only (*p-value* = 0.01). Moreover, mortality rate was increased in the methylated group. This finding is consistent with our previous finding that, patients with higher *LGALS12* expression have the better overall survival (El Leithy et al., 2015).

## Conclusion

The methylation pattern of the promoter region affects the expression only in galectin-12 but not in galectin-3. Our findings identify that hypermethylation of galectin-12 promoter is a common event in *de novo* adult AML. The abnormally hypomethylated and over-expressed galectin-12 cases had a relatively overall survival advantage. Galectin-3 downregulation is not a consequence of promoter methylation. However, intergenic and out of studied fragment DNA methylation cannot be excluded.

## Recommendation and future prospective

Galectin-12 promoter hypomethylation and relative over-expression showed an overall survival advantage in AML patients. The present study findings confirmation in other cohorts in the same and different populations is recommended. Consequently, future research for specifically targeting a hypomethylating therapy agent for methylated galectin-12 promoter region could be an advance in the treatment of AML. Furthermore, the prospective evaluation of the methylation status of the galectin-12 promoter region in AML patients will be highly recommended for adjusting the patient treatment protocol.

## Data Availability

The datasets presented in this study can be found in online repositories. The names of the repository/repositories and accession number(s) can be found in the article/supplementary material.
